# Supplementary figures and images for: Metagenomic analysis of nitrogen and methane cycling in the Arabian Sea oxygen minimum zone
Source: PeerJ. 2016 Apr 7;4:e1924. doi: 10.7717/peerj.1924 (PMC4830246; doi:10.7717/peerj.1924)

Assembly of 16S rRNA

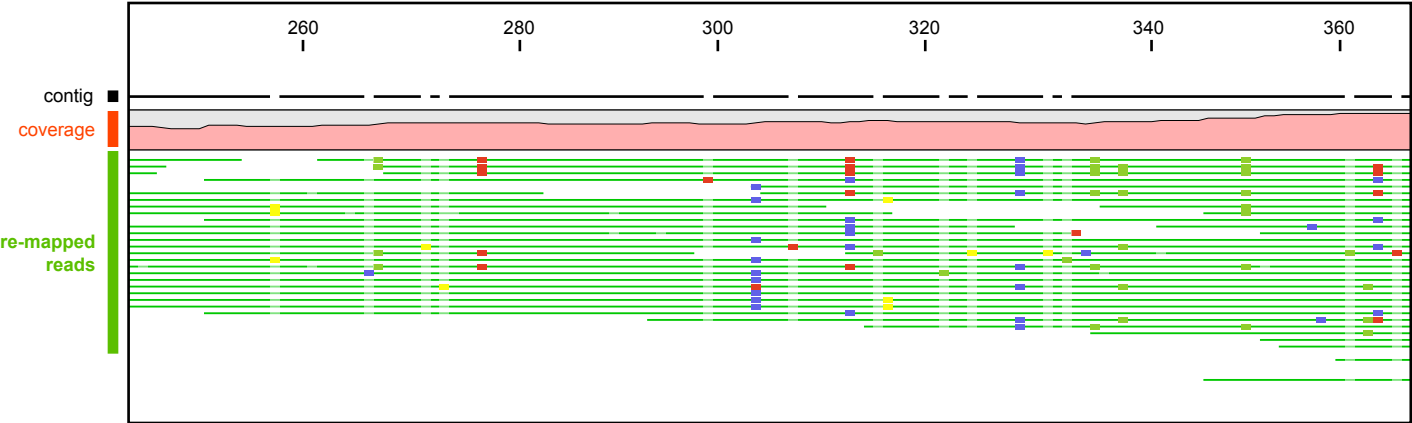

Supplement: Figure S1 — The black line represents the assembled contig sequence and green lines illustrate the re-mapped reads. Different colours within the re-mapped reads highlight positions with mismatches to the contig sequence. The overview shows that the contig sequence was not assembled from a single organisms, but is a hybrid sequence of various closely related Marine Group I Thaumarchaeota members. [file peerj-04-1924-s001.pdf]

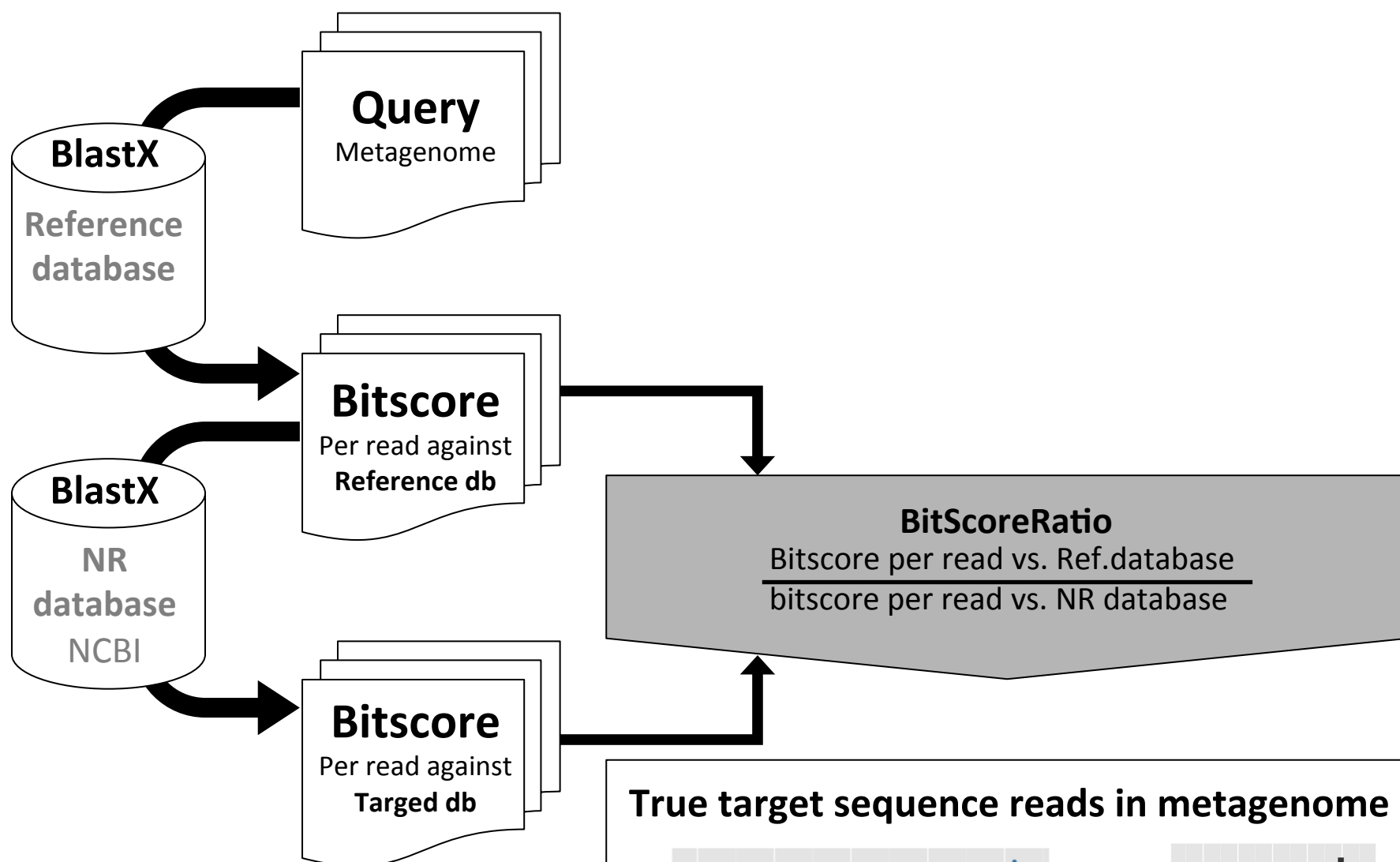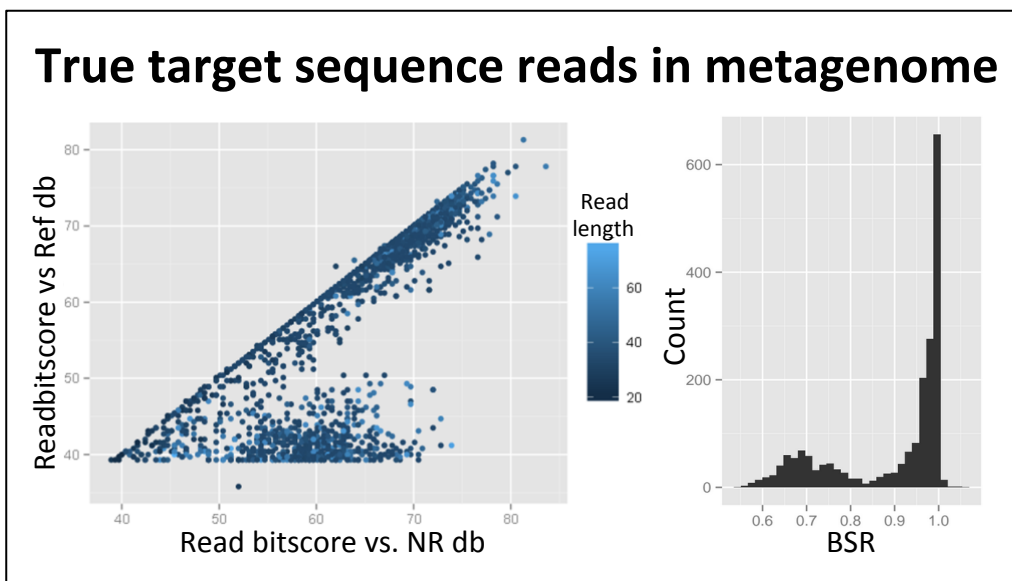

Supplement: Figure S2 — See Table 1 for individual bit-score used for each gene. [file peerj-04-1924-s002.pdf]

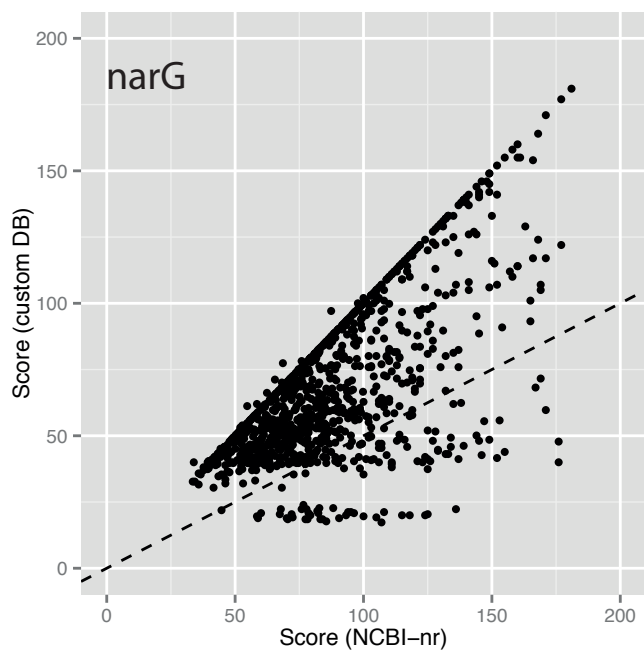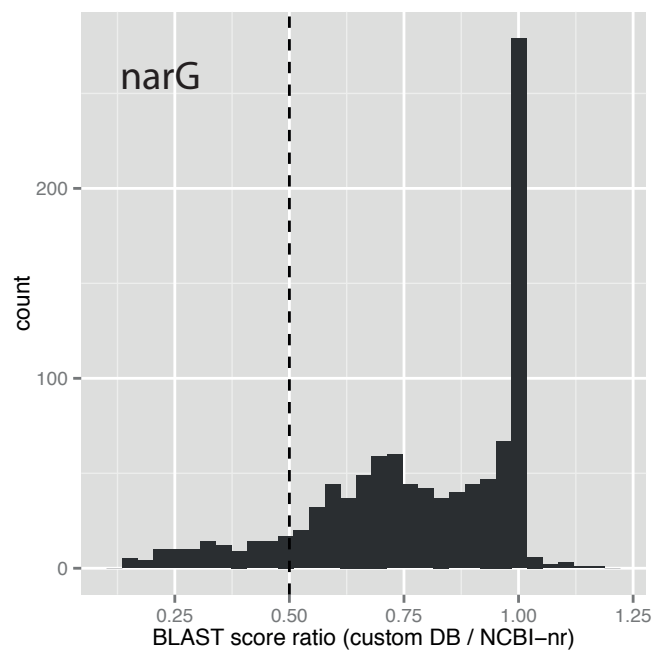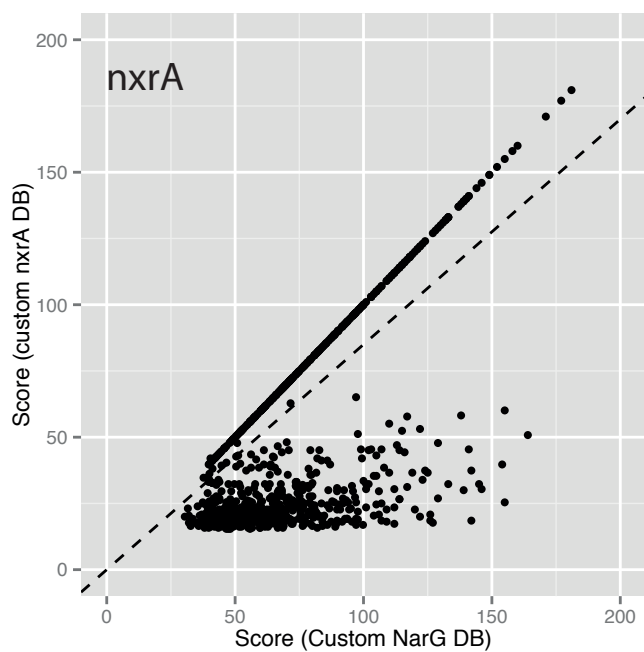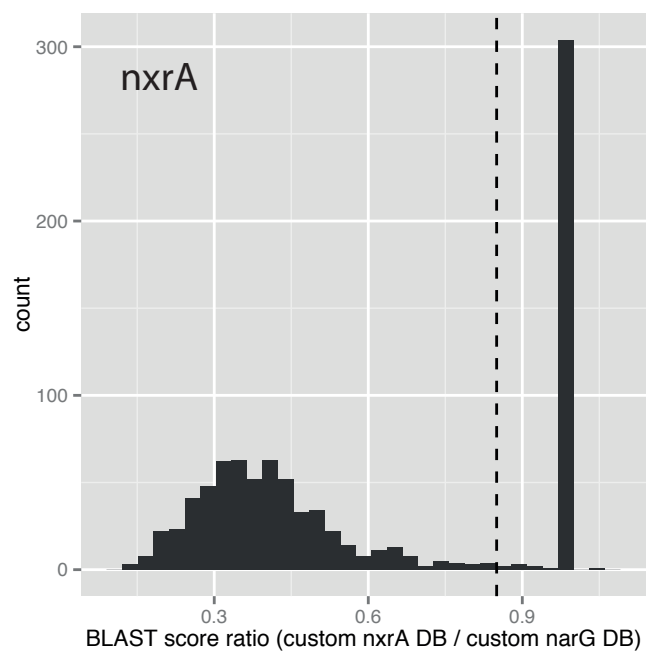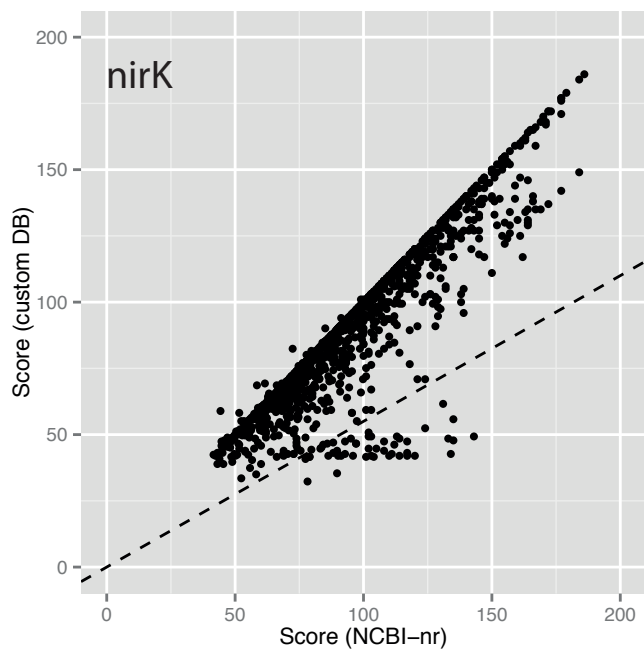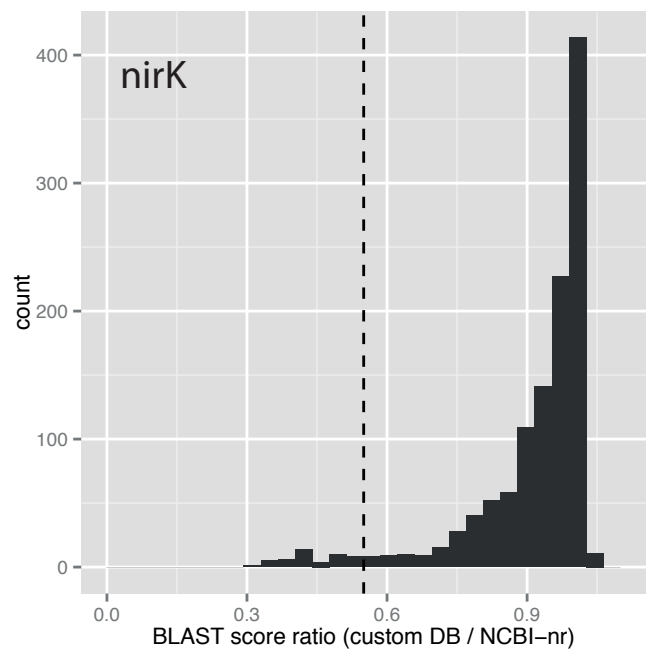

Supplement: Figure S3 — In the scatterplots on the left side, each point represents a read with a hit to our custom database. The dashed line represents the cut-off for inclusion in the analysis. The histograms on the right side represent a summary of the calculated BSR of all hits, with the dashed line representing the cut-off for inclusion in the analysis. [file peerj-04-1924-s003.pdf]

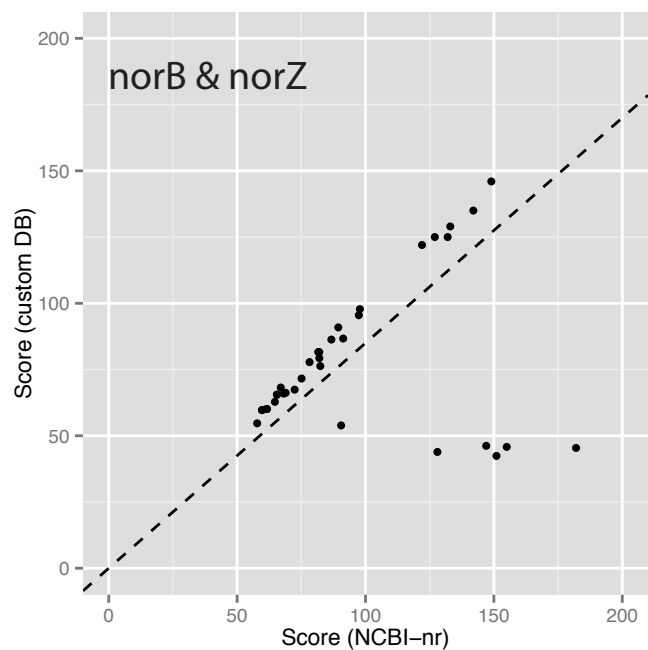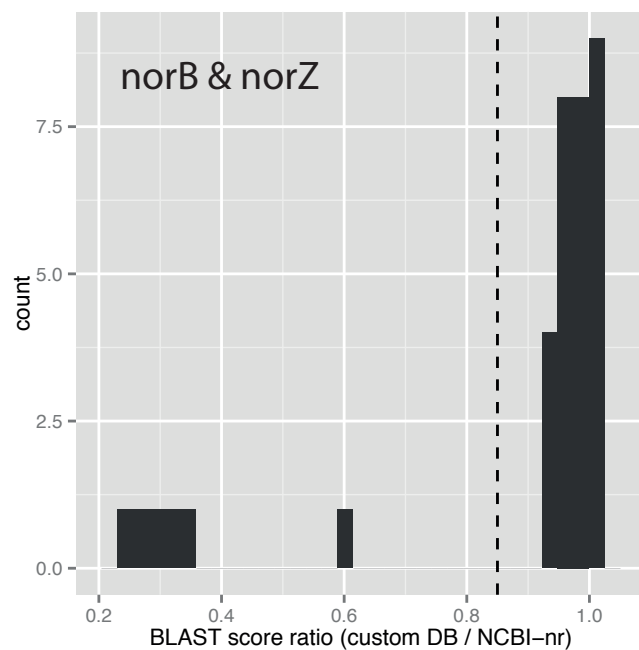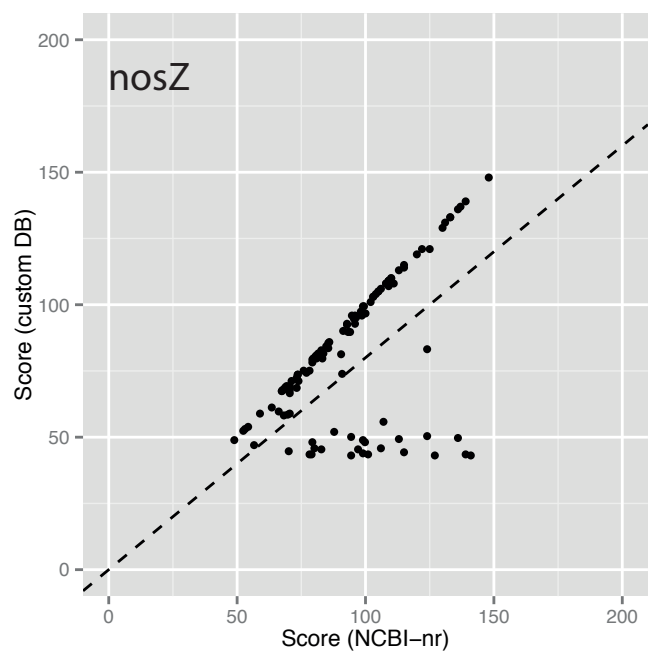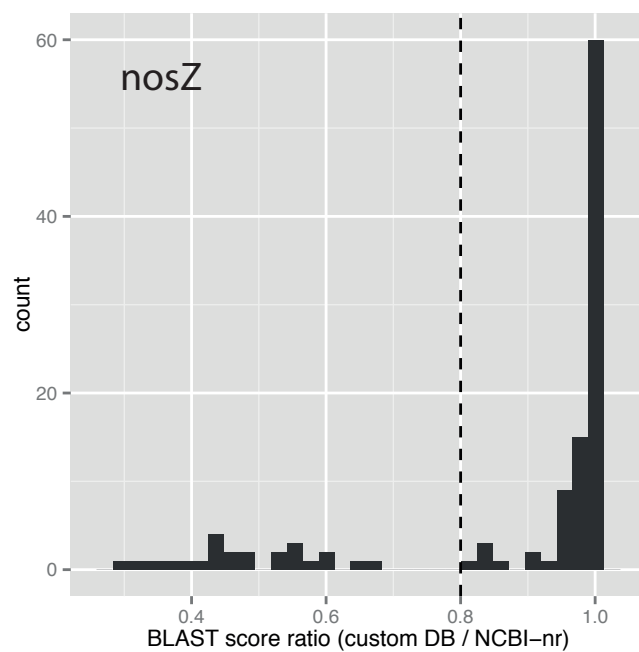

Supplement: Figure S4 — In the scatterplots on the left side, each point represents a read with a hit to our custom database. The dashed line represents the cut-off for inclusion in the analysis. The histograms on theright siderepresent a summary of the calculated BSR of all hits, with thedashed line representing the cut-off for inclusion in the analysis. [file peerj-04-1924-s004.pdf]

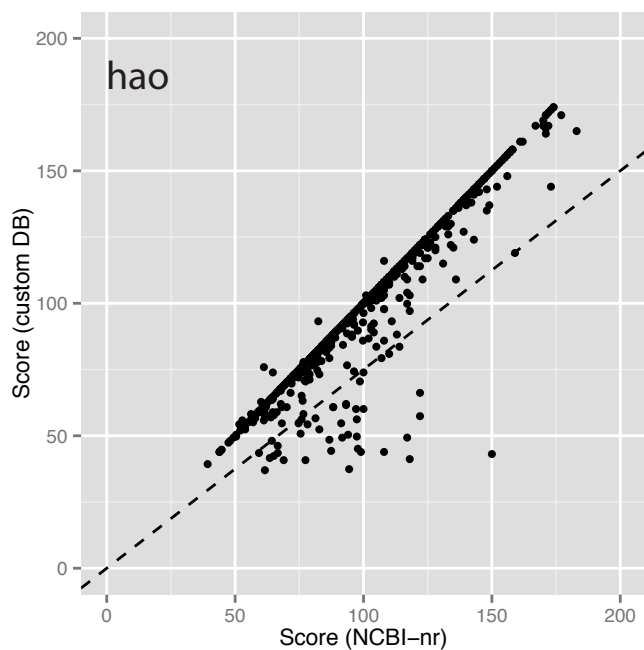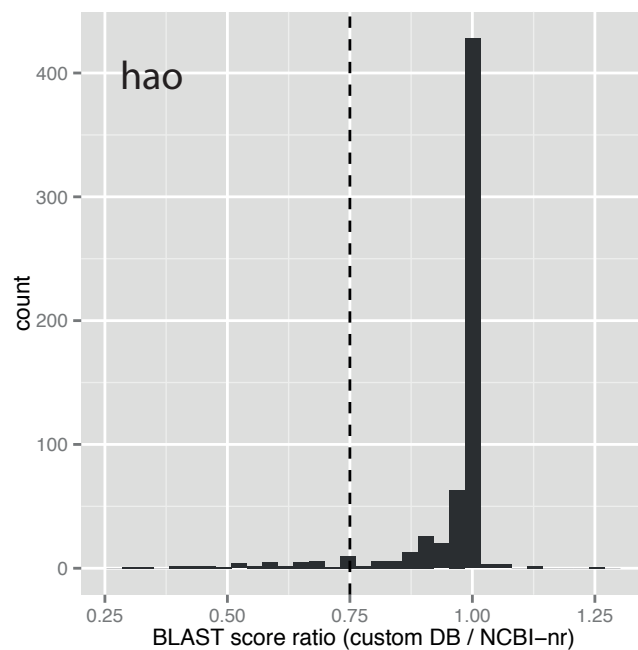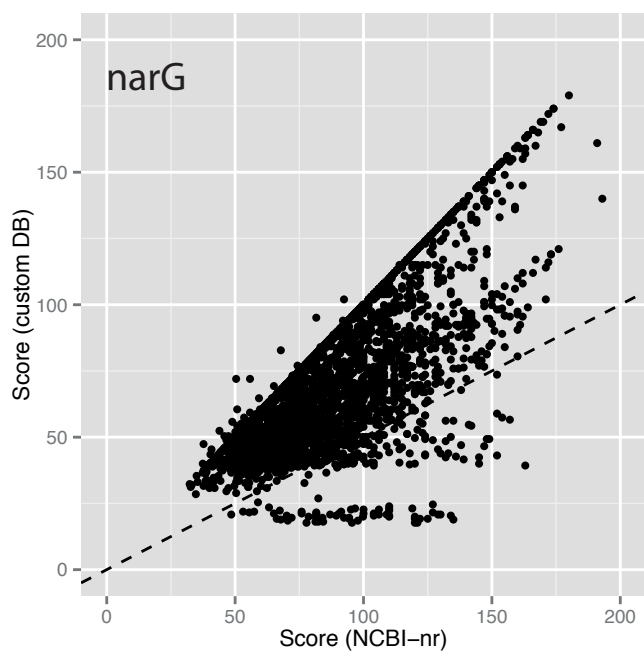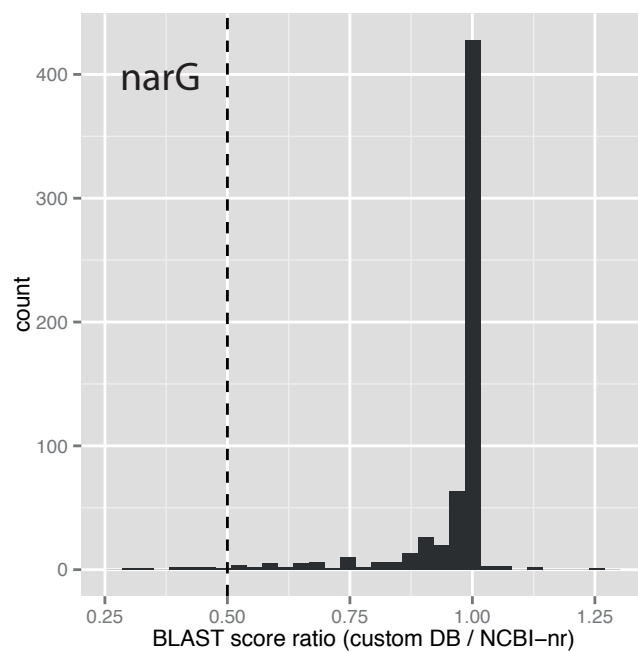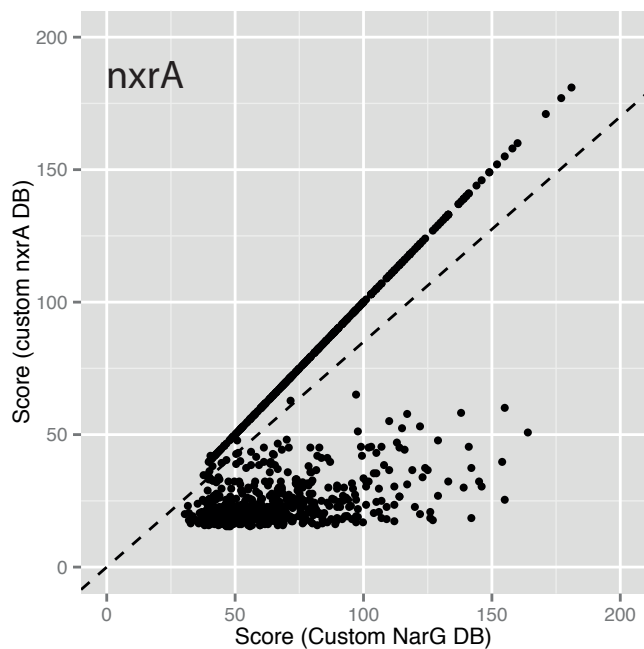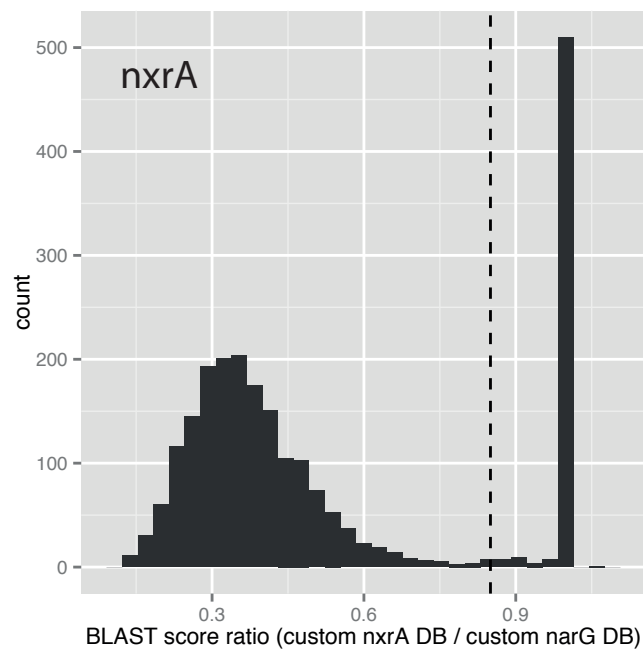

Supplement: Figure S5 — In the scatterplots on theleft side, each point represents a read with a hit to our custom database. The dashed line representsthe cut-off for inclusion in the analysis. The histograms on theright siderepresent a summary of the calculated BSR of all hits, with the dashedline representing the cut-off for inclusion in the analysis. [file peerj-04-1924-s005.pdf]

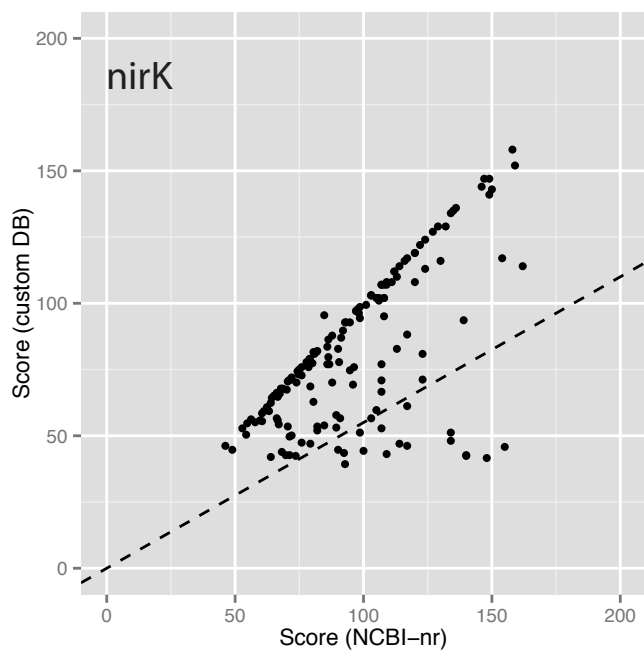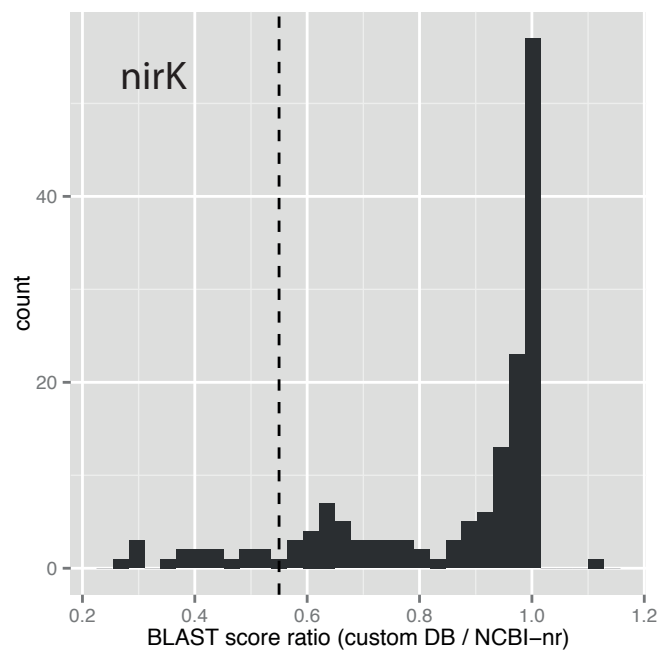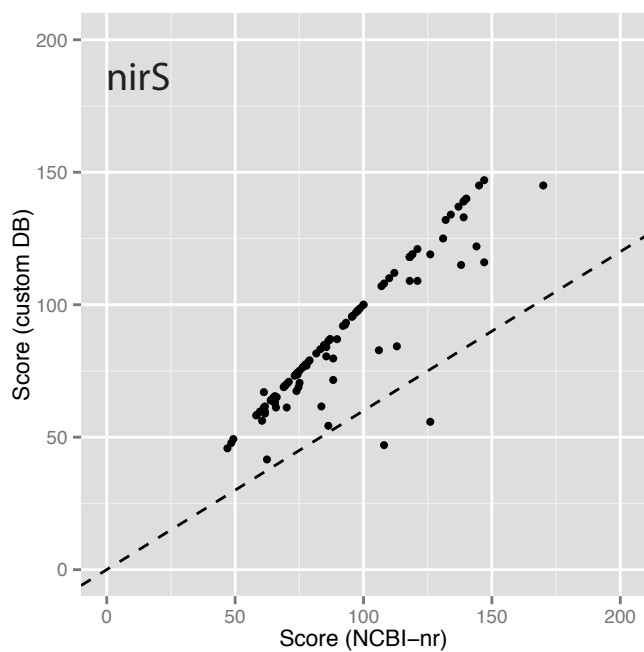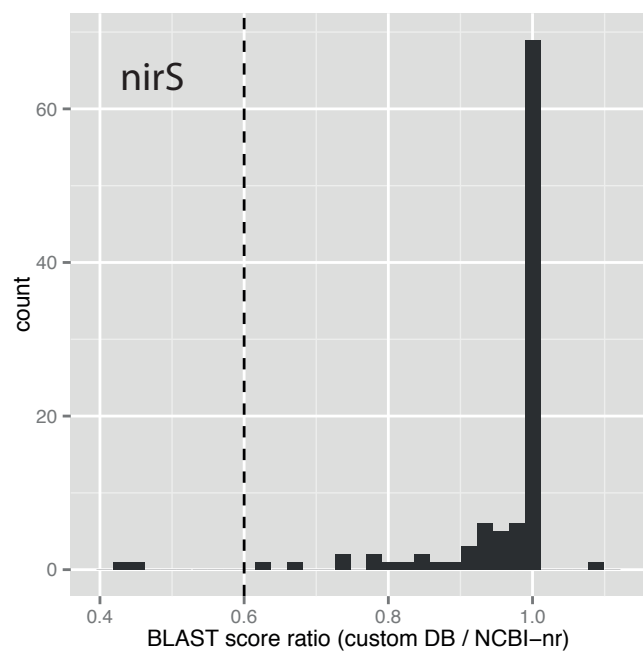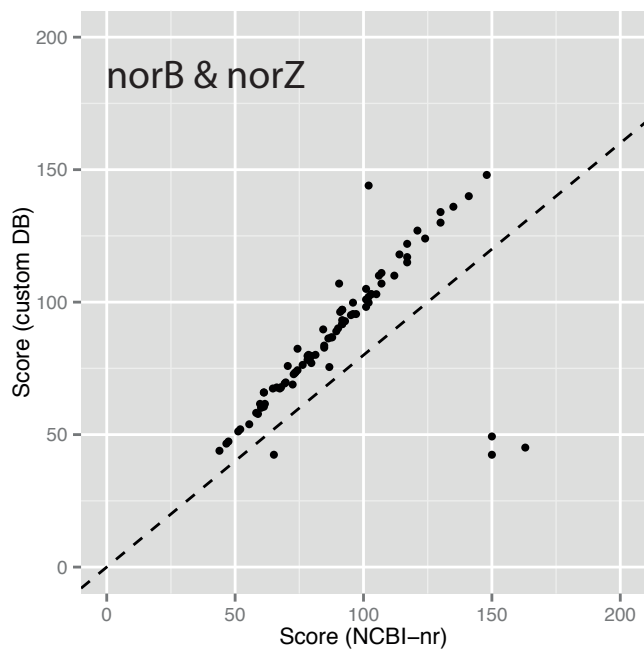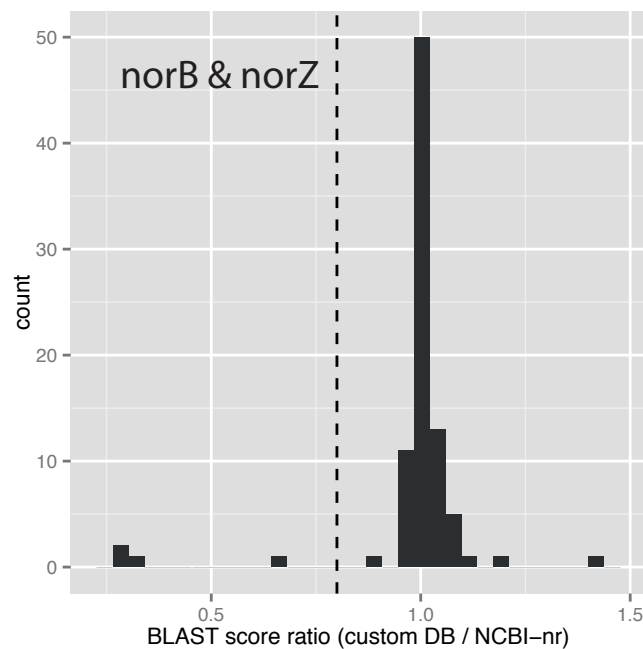

Supplement: Figure S6 — In the scatterplots on theleft side, each point represents a read with a hit to our custom database. The dashed linerepresents the cut-off for inclusion in the analysis. The histograms on theright siderepresent a summary of the calculated BSR of all hits, with thedashed line representing the cut-off for inclusion in the analysis. [file peerj-04-1924-s006.pdf]

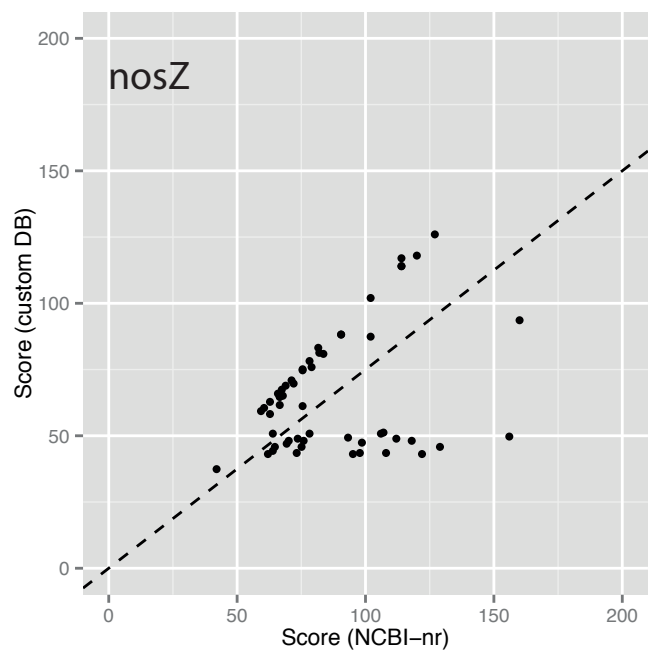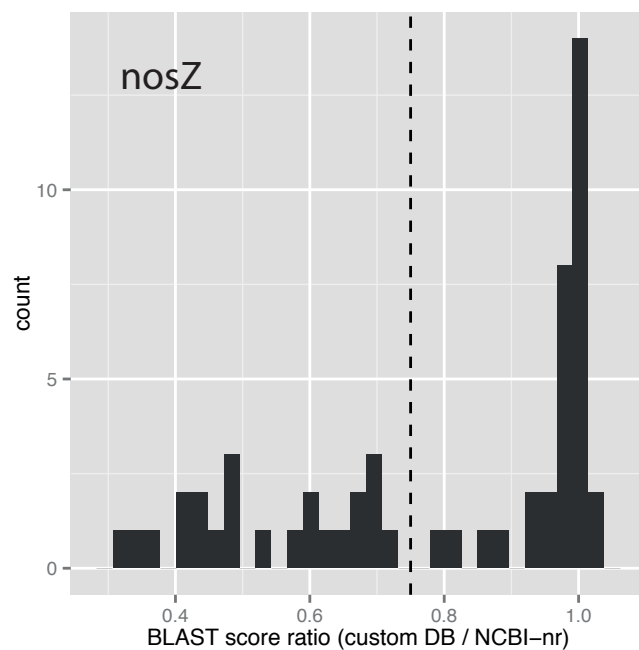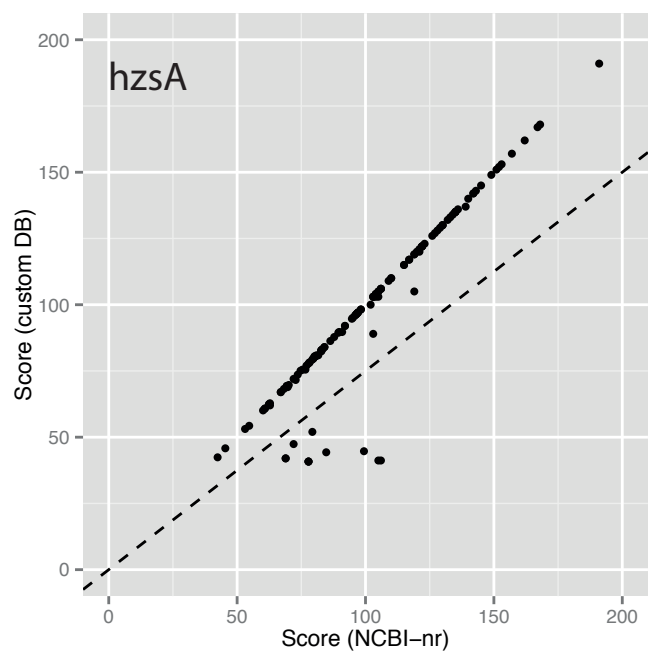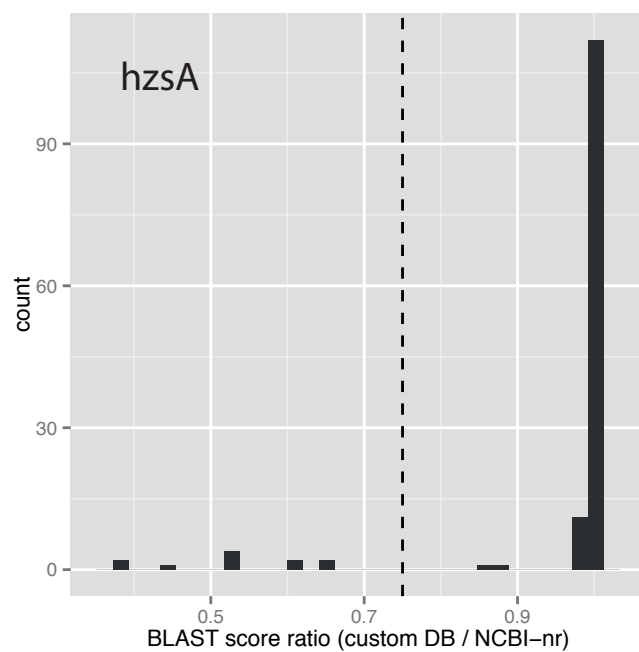

Supplement: Figure S7 — The dashed line represents the cut-off for inclusion in the analysis. The histograms on theright siderepresent a summary of the calculated BSR of all hits, with the dashedline representing the cut-off for inclusion in the analysis. [file peerj-04-1924-s007.pdf]
