# Supplementary material for: Metagenomic analysis of nitrogen and methane cycling in the Arabian Sea oxygen minimum zone
Source: PeerJ. 2016 Apr 7;4:e1924. doi: 10.7717/peerj.1924 (PMC4830246; doi:10.7717/peerj.1924)
Supplement: Table S1 — The data was taken from Villanueva et al. (2014). [file peerj-04-1924-s008.docx]

**Table S1.** Summary of the physicochemical conditions at the station PA2 (170m from the ocean surface) and PA5 (600m) of the Arabian Sea oxygen minimum zone. The data was taken from Villanueva et al. 2014.

| **Depth (m)** | **T (°C)** | **NH_4_^+^ (µM)** | **NO_2_^-^ (µM)** | **NO_3_^-^(µM)** | **HPO_4_^2-^ (µM)** | **Oxygen (µM)** |
| --- | --- | --- | --- | --- | --- | --- |
| 170 | 19.0 | 0.140 | 0.62 | 21.7 | 2.24 | 4.8 |
| 600 | 12.0 | 0.058 | 0.50 | 26.7 | 2.77 | 3.4 |
