# Supplementary material for: Metagenomic analysis of nitrogen and methane cycling in the Arabian Sea oxygen minimum zone
Source: PeerJ. 2016 Apr 7;4:e1924. doi: 10.7717/peerj.1924 (PMC4830246; doi:10.7717/peerj.1924)
Supplement: Supplementary file 3 [file peerj-04-1924-s009.odt]

Table S2. Number and taxonomic classification of reads affiliated with the aerobic part of the nitrogen cycling (see Figure 4) in the Arabian Sea metagenomes. For the comparisons of gene abundances in Figure 4, the numbers shown in this table were normalized according to gene length and metagenome size (PA2 metagenome: 3,254,623 reads, PA5 metagenome: 1,641,277 reads). The following formula was applied: Normalized read count=(number of mapped reads)*1000bp*1 million reads/[(length of gene)*(number of total reads in the metagenome)]. Taxonomic classification was performed using the lowest common ancestor algorithm implemented in the MEGAN software. Only phylogenetic groups accounting for more than 1% of the total community in at least one of the two datasets are listed.

| Taxonomy | 16S rRNA | nifH | amoA | hao | nxrA |
| --- | --- | --- | --- | --- | --- |
| PA2 | PA5 | PA2 | PA5 | PA2 | PA5 | PA2 | PA5 | PA2 | PA5 |
| alpha-Proteobacteria | SAR11 | 290 | 90 | 0 | 0 | 0 | 0 | 0 | 0 | 0 | 0 |
| Rhodobacteriales | 30 | 29 | 0 | 0 | 0 | 0 | 0 | 0 | 0 | 0 |
| Rhodospirillales | 30 | 24 | 0 | 0 | 0 | 0 | 0 | 0 | 0 | 0 |
| Gamma-Proteobacteria | Alteromonadales | 39 | 12 | 0 | 0 | 0 | 0 | 0 | 0 | 0 | 0 |
| SAR86 | 160 | 17 | 0 | 0 | 0 | 0 | 0 | 0 | 0 | 0 |
| SUP05 | 58 | 30 | 0 | 0 | 0 | 0 | 0 | 0 | 0 | 0 |
| Salinisphaerales | 32 | 21 | 0 | 0 | 0 | 0 | 0 | 0 | 0 | 0 |
| E01-9C-26 | 11 | 18 | 0 | 0 | 0 | 0 | 0 | 0 | 0 | 0 |
| delta-Proteobacteria | SAR324 | 102 | 34 | 0 | 0 | 0 | 0 | 0 | 0 | 0 | 0 |
| Bacteriodetes | Flavobacteriales | 41 | 6 | 0 | 0 | 0 | 0 | 0 | 0 | 0 | 0 |
| Planctomycetes | Brocadiales | 1 | 58 | 0 | 0 | 0 | 0 | 2 | 430 | 127 | 345 |
| Phycisphaerae | 2 | 12 | 0 | 0 | 0 | 0 | 0 | 0 | 0 | 0 |
| Verrucomicrobia | Arctic97B-4 | 22 | 3 | 0 | 0 | 0 | 0 | 0 | 0 | 0 | 0 |
| Deferribacteres | SAR406 | 172 | 220 | 0 | 0 | 0 | 0 | 0 | 0 | 0 | 0 |
| Nitrospinae | Nitrospina | 28 | 30 | 0 | 0 | 0 | 0 | 0 | 0 | 105 | 68 |
| Actinobacteria | Acidimicrobia | 65 | 27 | 0 | 0 | 0 | 0 | 0 | 0 | 0 | 0 |
| Chloroflexi | SAR202 | 16 | 27 | 0 | 0 | 0 | 0 | 0 | 0 | 0 | 0 |
| Dehalococcoidia | 0 | 18 | 0 | 0 | 0 | 0 | 0 | 0 | 0 | 0 |
| Thaumarchaeota | Marine Group I | 283 | 36 | 0 | 0 | 227 | 20 | 0 | 0 | 0 | 1 |
| Euryarchaeota | Marine Group II | 151 | 4 | 0 | 0 | 0 | 0 | 0 | 0 | 0 | 0 |
| Marine Group III | 29 | 5 | 0 | 0 | 0 | 0 | 0 | 0 | 0 | 0 |
| Halobacteria | 0 | 42 | 0 | 0 | 0 | 0 | 0 | 0 | 0 | 0 |
| Sum of remaining unclassified taxa | 460 | 344 | 0 | 3 | 1 | 0 | 3 | 45 | 66 | 75 |
